# Molecular Markers Reveal Epidemiological Patterns and Evolutionary Histories of the Human Pathogenic *Cryptococcus*

**DOI:** 10.3389/fcimb.2021.683670

**Published:** 2021-05-06

**Authors:** Nan Hong, Min Chen, Jianping Xu

**Affiliations:** ^1^ Department of Dermatology, Shanghai Key Laboratory of Medical Mycology, Changzheng Hospital, Naval Medical University, Shanghai, China; ^2^ Department of Burn and Plastic Surgery, Jinling Hospital, School of Medicine, Nanjing University, Nanjing, China; ^3^ Department of Biology, McMaster University, Hamilton, ON, Canada

**Keywords:** *Cryptococcus neoformans* species complex, *Cryptococcus gattii* species complex, genetic variation, gene flow, discrimination power, microevolution

## Abstract

The human pathogenic *Cryptococcus* species are the main agents of fungal meningitis in humans and the causes of other diseases collectively called cryptococcosis. There are at least eight evolutionary divergent lineages among these agents, with different lineages showing different geographic and/or ecological distributions. In this review, we describe the main strain typing methods that have been used to analyze the human pathogenic *Cryptococcus* and discuss how molecular markers derived from the various strain typing methods have impacted our understanding of not only cryptococcal epidemiology but also its evolutionary histories. These methods include serotyping, multilocus enzyme electrophoresis, electrophoretic karyotyping, random amplified polymorphic DNA, restriction fragment length polymorphism, PCR-fingerprinting, amplified fragment length polymorphism, multilocus microsatellite typing, single locus and multilocus sequence typing, matrix-assisted laser desorption/ionization time of flight mass spectrometry, and whole genome sequencing. The major findings and the advantages and disadvantages of each method are discussed. Together, while controversies remain, these strain typing methods have helped reveal (i) the broad phylogenetic pattern among these agents, (ii) the centers of origins for several lineages and their dispersal patterns, (iii) the distributions of genetic variation among geographic regions and ecological niches, (iv) recent hybridization among several lineages, and (v) specific mutations during infections within individual patients. However, significant challenges remain. Multilocus sequence typing and whole genome sequencing are emerging as the gold standards for continued strain typing and epidemiological investigations of cryptococcosis.

## Introduction

The human pathogenic *Cryptococcus* species are among the biggest causes of mobility and mortality in humans. Globally, there are ~223,100 cases of cryptococcal meningitis per year, primarily in HIV-infected patients, resulting in ~181,100 deaths (Rajasingham et al., 2017). Strains in two sibling species complexes, the *Cryptococcus neoformans* species complex (CNSC) and the *Cryptococcus gattii* species complex (CGSC), are the primary causative agents of cryptococcosis (Kwon-Chung et al., 2014; May et al., 2016). CNSC has a worldwide distribution, occurring naturally in the soil, avian excreta, and decayed wood inside trunk hollows of many tree species (Lazera et al., 2000; Randhawa et al., 2008; Taverna et al., 2020), and is responsible for over 80% of the global cryptococcal infections (Park et al., 2009). CGSC has been mainly reported from the decayed wood in trunk hollows of various trees in tropical and subtropical regions and is observed to cause diseases in apparently healthy individuals (Byrnes et al., 2009; Mcculloh et al., 2011; Chen et al., 2014).

Since the 1980s, several molecular typing methods have been developed to study the epidemiology and genetic diversity of these two species complexes (**Table 1**). These methods primarily assay DNA sequence variations among strains and lineages and include random amplified polymorphic DNA (RAPD), PCR-fingerprinting, amplified fragment length polymorphisms (AFLP), PCR-restriction fragment length polymorphisms (PCR-RFLP), single locus sequence typing (SLST), multilocus sequence typing (MLST), multilocus microsatellite typing (MLMT), and whole genome sequencing (Sidrim et al., 2010; Li et al., 2013; Cuomo et al., 2018). The pros and cons of each of these methods are briefly summarized in **Table 1**. Together, these methods have allowed the identifications of individual and/or groups of strains, clarified the phylogenetic diversity within and between the two species complexes, revealed both recent and ancient hybridizations among lineages, and identified both broad geographic patterns of strain genotype distributions and local transmissions and microevolutions. In the sections below, we briefly describe each molecular typing method, point out their advantages and disadvantages, and summarize the main epidemiological findings based on each method. We also discuss how the methods have enabled other types of studies on the origins, virulence, and drug resistance in this important group of fungal pathogens.

**Table 1:** Comparison of major molecular methods used for typing the human pathogenic *Cryptococcus*.

Methods	Genes/Molecules	Cost	Specificity	Convenience	Reproducibility	Discriminating Power	Resolution	First report
Serotyping	Capsular surface antigen	Medium	Medium	Medium	High	Low	Five serotypes A, B, C, D, AD	Kwon-Chung et al., 1982
RAPD	Random sequence in genome	Low	Low	High	Low	High	Strain-level	Welsh and McClelland, 1990
PCR fingerprinting	Random sequence in genome	Low	Low	High	Medium	Medium	Medium	Jeffreys et al., 1985
AFLP	Random sequence in genome	Medium	Low	Low	Medium	High	High	Vos et al., 1995
MLMT	Specific loci in genome	Medium	High	Medium	High	Medium to high depending on loci	Medium to high depending on loci	De Valk et al., 2007
MLST	Specific loci in genome	Medium	High	Medium	High	Medium to high depending on loci	Medium to high depending on loci	Xu et al., 2000b
MALDI-TOF MS	Whole-cell proteome	Medium	Low	Medium	Medium	Medium to high	Medium to high	Firacative et al., 2012
WGST	Whole genome sequence	High	High	Low	High	High	High	Fraser et al., 2005

## Serotyping Methods

Serotypes of CNSC and CGSC reflect the antigenic differences resulting from variation in capsular polysaccharides (Cherniak and Sundstrom, 1994). A diagnostic medium containing L-canavanine, glycine and bromothymol blue (CGB test) was first used to identify two species complexes including four serotypes: CNSC for serotypes A and D, and CGSC for serotypes B and C (Kwon-Chung et al., 1982). However, it was reported that a positive CGB reaction alone was not sufficient for accurate serotyping (Khan et al., 2003). Subsequently, antisera raised from strains of four different serotypes were developed into a commercial capsular agglutination test kit to identify cryptococcal serotypes. Based on the capsular agglutination reactions, an additional serotype AD that reacted with both serotypes A and D antisera was identified (Belay et al., 1996).

Serotyping was a quick and widely used epidemiological tool in cryptococcal research until the early part of the 21^st^ century. Those studies identified that globally, the most frequent serotype in both environmental and clinical samples belonged to serotype A. In contrast, other serotypes showed more patchy distributions. For example, serotypes B and C were mainly found in tropical and subtropical regions while serotype D was mainly found in southern Europe (Dromer et al., 1996; Tortorano et al., 1997; Viviani et al., 2006). Further research revealed that different serotypes each had unique microbiological and biochemical characteristics (Nakamura et al., 1998). However, there were several limitations in using serotyping as the only strain typing method. For example, there were natural isolates without any capsular polysaccharides and thus were not reacting with any of the specific antisera and were deemed not typable (Fromtling et al., 1982; Kwon-Chung and Rhodes, 1986). In addition, the discrimination power of serotyping method is comparatively low which makes it unable to meet the demand of modern epidemiological studies of CNSC and CGSC.

At the turn of the century, DNA sequence analyses revealed significant divergence among strains and serotypes of the human pathogenic *Cryptococcus*. Those sequence analyses revealed restriction site polymorphism within specific genes that could be readily used to distinguish strains of different serotypes. For example, PCR amplification of a fragment of *CAP59*, a gene required for capsule biosynthesis, followed by restriction enzyme digest, enabled successful identification of the serotypes of CNSC and CGSC (Nakamura, 2001; Enache-Angoulvant et al., 2007). Later on, a multiplex PCR based on a set of four primers was created for serotype identification (Ito-Kuwa et al., 2007). These PCR-based methods showed high concordance with immunological serotyping methods. The cost-effectiveness and convenience of PCR-based methods for serotype identification resulted in the disuse of antisera serotyping kits as a common tool for epidemiological studies.

## Multilocus Enzyme Electrophoresis

Multilocus enzyme electrophoresis (MLEE) is a molecular typing method that detects differences in gel electrophoretic migration patterns of enzymes. The differences in mobility through gel matrixes are due to non-synonymous substitutions in genes encoding for enzymes. Since many enzymes are polymorphic among individuals in most species, MLEE was a very popular molecular typing method from the 1960s to the 1990s for assaying genetic variation in a diversity of organisms, from bacteria to fungi, plants and animals, including humans. For each metabolic enzyme, each individual haploid organism has a specific amino acid sequence, exhibiting a specific mobility during gel electrophoresis. For diploid organisms, each individual would typically contain two copies of each gene. If the two copies are identical to each other within an individual, the individual is called a homozygote at the locus and only one enzyme band would be found during gel electrophoresis for the specific enzyme in this individual. In contrast, if the two copies of a metabolic enzyme have different amino acid sequences that change the mobility of the enzyme, the individual is called a heterozygote at the locus and two enzyme bands would be found during gel electrophoresis for the specific enzyme in the individual. After electrophoresis, specific colorimetric substrates are added to the gel to detect the mobility of individual enzymes. The mobility patterns of multiple enzymes constitute the individual organism’s electrophoretic profile. MLEE offers higher discriminatory power than serotyping for detecting clones of many human microbial pathogens.

In 1995, Brandt et al. investigated the usefulness of MLEE in subtyping human pathogenic *Cryptococcus* clinical isolates (Bertout et al., 1999). They assayed the following 10 enzymes: alcohol dehydrogenase (EC 1.1.1.1), glucose 6-phosphate dehydrogenase (EC 1.1.1.49), 6-phosphogluconate dehydrogenase (EC 1.1.1.44), glutamate dehydrogenase (EC 1.4.1.4), glutamate oxaloacetic transaminase (EC 2.6.1.1), malate dehydrogenase (EC 1.1.1.37), phosphoglucose isomerase (EC 5.3.1.9), phosphoglyceromutase (EC 2.7.5.1), phenylalanine-leucine peptidase (EC 3.4.X.X), and leucine-alanine peptidase (EC 3.4.X.X). All 10 enzymes were found to be polymorphic in the analyzed *Cryptococcus* population. Based on the electrophoretic patterns of these 10 enzymes, they separated 344 isolates into 19 MLEE electrophoretic types (ETs): serotype A strains were grouped into 10 ETs; and three ETs each were found for strains of serotype D, serotype AD, and serotype B respectively (Brandt et al., 1995). The applications of the MLEE method allowed them to infer the epidemiological patterns of cryptococcal infections in four U.S. metropolitan areas between 1992 to 1994 (Brandt et al., 1995). Specifically, though some of the ETs were broadly distributed, they found several geographic specificities. For example, ET-1, the predominant ET throughout the US, was recovered in significantly greater proportion from Atlanta (Georgia), Houston (Texas), and all major metropolitan areas of Alabama than from San Francisco (California). In contrast, ET-2 and ET-7 (serotype AD) isolates were recovered predominantly from San Francisco (Brandt et al., 1995). Multiple isolates from the same patient always had the same ET, consistent with the stability of these markers and the persistence of cryptococcal strains in these patients. Furthermore, no significant difference in ET type distribution was found when isolates from HIV-infected patients were compared with those without HIV-infections, indicating no evidence of selection by the host or the pathogen in the genotype of cryptococcal strains causing cryptococcosis among patient groups in the US (Brandt et al., 1995).

While MLEE played a major role for studying a wide variety of practical and theoretical questions relating to epidemiology, population genetics, and systematics of microbial pathogens, it has ceased to be a routine strain typing technique of clinical or environmental microorganisms, including those of the human pathogenic *Cryptococcus*. Several reasons have likely contributed to its limited use, including: (i) technical complexity of MLEE; (ii) difficulties in comparing mobility results among laboratories; (iii) inability to assess nucleotide substitutions that do not cause amino acid changes (synonymous substitutions) due to redundancy in genetic code; and (iv) inability to assess amino acid substitutions that do not alter the overall charge or obvious molecular weight among alleles. Both (iii) and (iv) will cause different alleles with either different DNA sequences and/or different amino acid sequence to have the same or indistinguishable electrophoretic mobilities on gels. In addition, MLEE can only detect polymorphisms within the coding region of each gene. Mutations in promoter regions or introns of a gene cannot be assayed by MLEE. Methods that directly assay DNA sequence variations could help eliminate the limitations intrinsic to MLEE.

## Electrophoretic Karyotyping

Karyotyping is a classical technology by which photographs of chromosomes are taken in order to determine the chromosome complement of an individual organism, including the number, size, and staining patterns of chromosomes. Any abnormalities in the number, size, and staining patterns of chromosomes among individuals can all be theoretically determined. In humans, chromosomal karyotyping is most commonly used for prenatal screening and to diagnose chromosomal abnormalities that cause infertility. In fungi, due to the small sizes of their chromosomes, karyotyping is not accomplished by staining and microscopy but by pulsed-field gel electrophoresis (PFGE). The application of PFGE for studying the human pathogenic *Cryptococcus* began in the early 1990s. Indeed, it was commonly used as an epidemiological tool for clinical and environmental isolates of CNSC and CGSC during the period of 1990s to 2000s (Kwon-Chung et al., 1992; Boekhout et al., 1993; Perfect et al., 1993; Saracli et al., 2006; Esposto et al., 2009). For example, Boekhout et al. reported that the electrophoretic karyotypes of CNSC and CGSC consist of seven to fourteen bands of chromosomal DNA, and that there was no obvious correlation between chromosomal number and serotypes, geographic origin or ecological habitat (Boekhout et al., 1993). In addition, while differences among isolates were observed, the karyotype patterns of individual CNSC or CGSC isolates were shown to be stable during both *in vitro* passage and *in vivo* infections (Boekhout et al., 1993). Those analyses revealed no specific pattern of karyotypes of CNSC or CGSC isolates being associated with site of infection [e.g., isolates from cerebral spinal fluid (CSF) vs blood] or with the hosts’ underlying conditions (e.g., isolates from HIV-infected patients compared with patterns in non-HIV-infected patients) (Perfect et al., 1993). Moreover, results of PFGE karyotyping supported the hypothesis that serotype AD isolates of CNSC often contained most chromosomal complements of both serotypes A and D strains (Esposto et al., 2009). In addition, PFGE karyotyping helped provide estimates of genome size and chromosome numbers in individual strains. For example, using PFGE, Perfect *et al*. provided the first estimate of the genome size of CNSC as between 15 and 17 Mb, with the number of chromosomes ranging between 10 and 12 (Perfect et al., 1993). Later, also using PFGE, Wickes *et al*. derived larger and more accurate genome size estimates at 21 to 24.5 Mb, with 13 chromosomes on average in CGSC and 12 chromosomes in CNSC (Wickes et al., 1994).

Notably, PFGE revealed significant electrophoretic karyotype polymorphisms among clinical and environmental isolates of CNSC and CGSC (Kwon-Chung et al., 1992; Perfect et al., 1993). For instances, Currie *et al*. reported 18 different karyotypes among 25 environmental and clinical isolates of CNSC from New York, USA (Currie et al., 1994). Similarly, six and three karyotypes were identified among 21 clinical isolates and eight environmental isolates of CNSC from Nagasaki, Japan (Yamamoto et al., 1995). In the study by Perfect *et al*. (Perfect et al., 1993), 41 different karyotypes were found among 46 clinical or environmental CNSC or CGSC isolates. Similarly, a high karyotype polymorphisms were found by Dromer et al. (Dromer et al., 1994) who reported 39 different karyotypes among 40 clinical isolates of CNSC (serotype D). Further, Perfect et al. noted that PFGE karyotyping could be used to exclude the possibility of nosocomial spread of CNSC in one clinical situation and supported relapse in two other cases due to its variable chromosome sizes between isolates (Perfect et al., 1993). Together, these results indicated the PFGE karyotyping can be a powerful method for discriminating strains and for epidemiological studies of CNSC and CGSC.

Even in the omics era, the PFGE karyotyping can still be a useful tool for genetic and epidemiological studies on CNSC and CGSC, particularly for determining chromosomal structural changes associated with environmental outbreaks or distinguishing the clinical isolates between relapse and reinfection. For example, drug resistance can be caused by duplication of whole chromosomes or chromosomal segments. Such duplications can often be detected through PFGE while other DNA sequence – based methods may miss such genetic changes. Together with other methods such as targeted gene sequencing or whole genome sequencing, PFGE will continue to help understand the epidemiology and evolution of CNSC and CGSC in the future.

## Restriction Fragment Length Polymorphism

Restriction fragment length polymorphism (RFLP) is a classic molecular genotyping technique originally developed for genetic studies and for constructing restriction maps of large DNA molecules such as plasmids and mitochondrial genomes. RFLP has been broadly employed for mapping human disease genes beginning in the late 1970s (Botstein et al., 1980). The RFLP-based molecular markers provide an ability to detect DNA fragments of different lengths after digestion of DNA samples from various sources by restriction endonucleases. Depending on our prior information, RFLP can be detected using different approaches. When the DNA sequence of the target gene is not known and PCR amplification is not possible, RFLP is typically determined based on the restriction digest of whole-genome DNA, followed by agarose gel electrophoresis, Southern blotting, hybridization of the labeled target DNA molecule to the Southern blot, and visualization of the hybridization products through autoradiography (Grover and Sharma, 2016). Although the procedure described above for assaying RFLP is reliable for distinguishing different genotypes and does not require knowledge of the genome sequence (Gebhardt et al., 1989), the entire procedure is very time-consuming and requires special training and equipment.

When the specific DNA sequence containing the restriction polymorphic site is known, PCR primers can be designed and RFLP can be effectively combined with PCR to quickly assay RFLP for many samples. For the human pathogenic *Cryptococcus*, Xu et al. in 2000 identified an RFLP within the mitochondrial large ribosomal subunit that distinguished serotypes A and D strains (Xu et al., 2000a). After 2001, Velegraki et al., Xu et al., Meyer et al. and Latouche et al. successively used PCR-RFLP in molecular epidemiological studies of clinical isolates of CNSC and CGSC, which targeted different genes including orotidine monophosphate pyrophosphorylase gene (*URA5*) (Velegraki et al., 2001; Xu, 2002; Meyer et al., 2003), the mitochondrial large ribosomal subunit (Velegraki et al., 2001; Xu, 2002; Meyer et al., 2003), and phospholipase B gene (*PLB1*) (Latouche et al., 2003). Commendably, the RFLP assays clustered CNSC and CGSC isolates into eight major molecular types, which have since found a good concordance with results of serotyping, PCR fingerprinting, and multilocus sequence typing, and whole-genome sequencing (please see below) of isolates of CNSC and CGSC. Additional PCR-RFLP markers based on other genes such as *CAP1* and *GEF1* allowed simultaneous identification of both the molecular type and mating type of CNSC and CGSC (Feng et al., 2008). Because of the ease of application, the PCR-RFLP markers will continue to be used for targeted epidemiological studies and genetic analyses of the human pathogenic *Cryptococcus*.

## Random Amplified Polymorphic DNA

PCR-fingerprinting is a series of PCR-based techniques using arbitrary primers to amplify regions of genomes. These techniques are versatile for detecting DNA sequence polymorphisms for a variety of purposes, including genetic mapping, phylogenetics, and molecular epidemiology (Welsh and Mcclelland, 1990; Williams et al., 1990). Among the PCR-fingerprinting techniques, random amplified polymorphic DNA (RAPD) is a molecular marker technique that uses a single short arbitrary oligonucleotide primer (generally 10 bp) to randomly amplify DNA fragments within individual genomes (Welsh and Mcclelland, 1990; Williams et al., 1990). Because the single primer is short, there are potentially many regions in the genome with similar or identical sequences that the primer can anneal to and amplify. Consequently, RAPD can potentially generate a large number of amplified fragments from different parts of the genome. Polymorphisms among individuals are generated when there are differences among individuals in nucleotide sequences at the primer sites and/or when there are insertions/deletions within the regions of DNA flanked by the primer recognition sites. The polymorphism information on target genomic DNA can be generated independent of any prior knowledge of the target DNA sequence. RAPD was broadly used in the early 1990s (Williams et al., 1990; Hadrys et al., 1992) when no whole-genome sequence was available. An appropriate primer may yield distinctive RAPD patterns of DNA fragments among species and strains. Most studies using RAPD markers for genotyping employ multiple primers, with one in each PCR. Together, a large number of polymorphic DNA bands can be generated and detected through agarose gel electrophoresis to distinguish strains. However, because of the short primer length and low annealing temperature, RAPD is sensitive to minor variations in many factors such as annealing temperature, buffer solution, template DNA concentration, and the PCR machine used. Consequently, it’s often necessary to standardize the procedures such as the quality and quantity of genomic DNA (Koebner, 1995; Mohan et al., 1995) and the specific PCR instrument used (Schierwater and Ender, 1993) in order to ensure reproducibility of RAPD results (Williams et al., 1990; Hadrys et al., 1992).

The RAPD technique was employed for several molecular epidemiological studies on CNSC and CGSC, using a series of primer combinations in the 1990s (Chen et al., 1996; Sorrell et al., 1996; Chen et al., 1997). Brandt et al. compared RAPD and MLEE and found they each had advantages, resolving the relationships among strains of CNSC and CGSC to different degrees (Brandt et al., 1995). Ruma et al. distinguished CNSC isolates between serotypes A, D or AD, and revealed further differentiation among strains within each serotype using seven RAPD primers (Ruma et al., 1996). In their study, four RAPD profiles were clearly distinguished within CGSC, among which two primers 5SOR and CNl differentiated their collection of CGSC isolates between serotypes B and C (Ruma et al., 1996). With primers ERICl and ERIC2, Boekhout et al. also reported that the RAPD technique could distinguish CNSC and CGSC isolates at the level of serotypes (Boekhout et al., 1997). Furthermore, RAPD markers have been successfully used in combination with other molecular typing methods. For example, Boekhout et al. showed that RAPD in combination with PFGE karyotyping were more useful in epidemiological studies of CNSC and CGSC than either technique alone (Boekhout et al., 1997). In other studies, the combination of long primer PCR-fingerprinting and RAPD revealed abundant genetic diversity of more than 400 isolates of CNSC and CGSC in two studies (Sorrell et al., 1996; Meyer et al., 1999), clustering the CNSC and CGSC isolates into four major groups, respectively.

**Figure 1:**
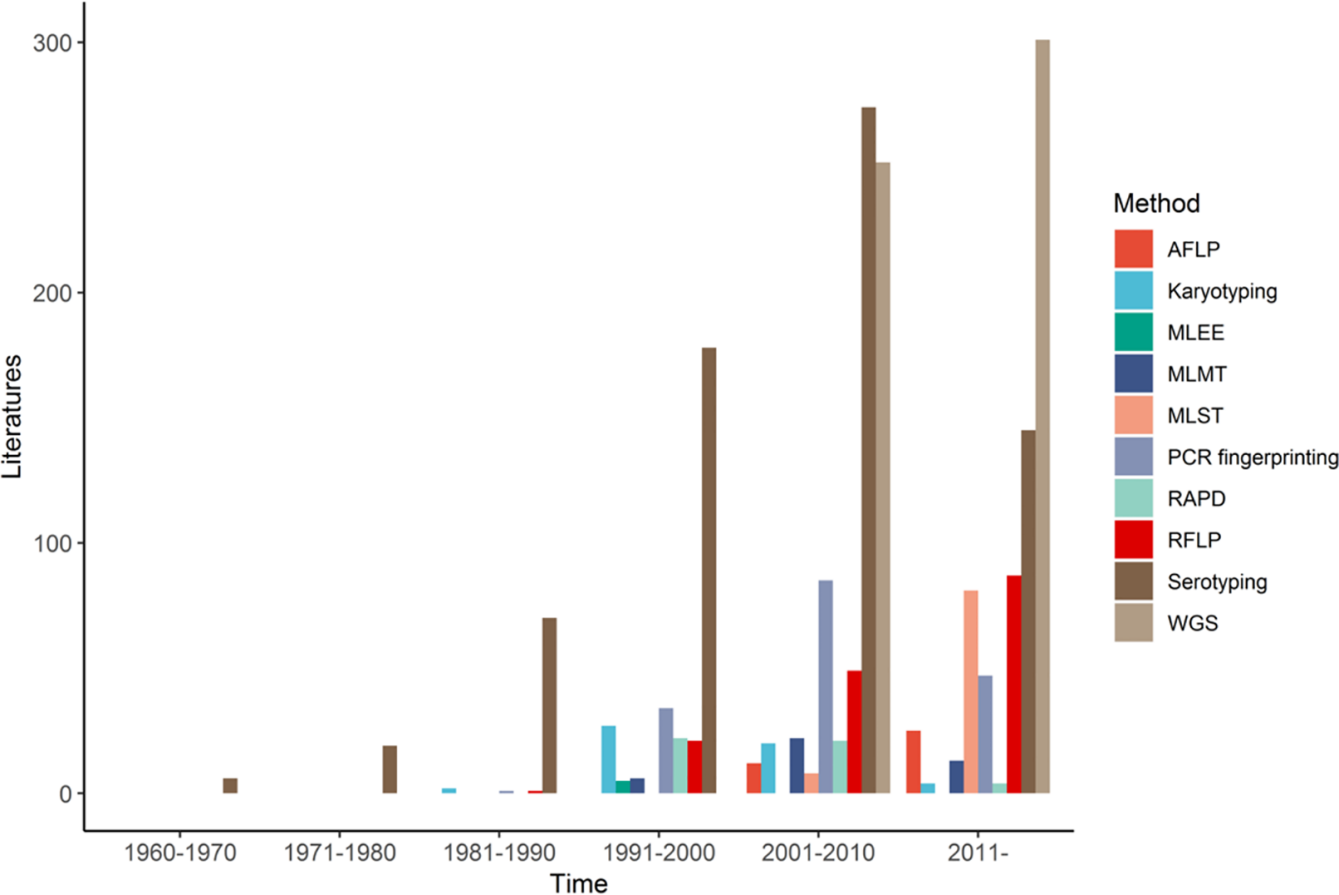
Number of publications using different molecular typing methods for studying the human pathogenic *Cryptococcus* from 1960 to 2021, by decade.

In general, while RAPD markers have generated useful information for epidemiological studies on CNSC and CGSC, its high sensitivity to experimental conditions and low reproducibility among experiments make comparing results among labs difficult (Williams et al., 1990; Hadrys et al., 1992). Indeed, there has been a continuous decline in using RAPD markers alone for epidemiological studies on CNSC and CGSC (and other organisms) since the early 1990s (**Figure 1**).

## PCR-Fingerprinting

As described in the last section, PCR fingerprinting is a common strain typing technique, first described in 1985 (Jeffreys et al., 1985). Different from RAPD, this strain typing technique typically refers to the detection of hypervariable repetitive sequences (minisatellite and microsatellite or simple repetitive DNAs) using specific oligonucleotides individually as PCR primers. These oligonucleotides were originally designed as hybridization probes for classical DNA fingerprinting experiments through Southern hybridization (Meyer et al., 1993a; Meyer et al., 1993b). Since 1993, Meyer et al. started to use a series of oligonucleotides hybridization probes such as (CA)_8_, (CT)_8_, (CAC)_5_, (GTG)_5_, (GACA)_4_, (GATA)_4_, and the phage M13 core sequence (5’-GAGGGTGGXGGXTCT-3’) as primers for PCR fingerprinting for strain typing and for molecular epidemiological analyses of fungal pathogens, including the human pathogenic *Cryptococcus* (Meyer et al., 1993a; Meyer et al., 1993b; Meyer and Mitchell, 1995). Because of the long primers and more stringent PCR amplification conditions, DNA fingerprints produced by PCR using primers such as (GTG)_5_, (GACA)_4_, or the M13 core sequence were much more reproducible than RAPD. Indeed, PCR fingerprinting reliably and successfully differentiated several lineages within CNSC and CGSC; with many isolates showing unique PCR fingerprint patterns (Meyer et al., 1993b). Since then, primers (GTG)_5_, (GACA)_4_, and the M13 core sequence have been widely recognized as standard PCR fingerprinting primers for studies on CNSC and CGSC (Meyer et al., 1993b; Meyer and Mitchell, 1995; Cogliati et al., 2000; Meyer et al., 2003). For example, in 1999, Meyer et al. used PCR fingerprinting to genotype 356 clinical isolates of CNSC and CGSC from around the globe (Meyer et al., 1999). They clustered these isolates into eight major molecular types, namely VNI (Serotype A), VNII (Serotype A), VNIII (Serotype AD), and VNIV (Serotype D) in CNSC; VGI, VGII, VGIII, and VGIV in CGSC (Meyer et al., 1999). These molecular types were later used for higher level taxonomy. Additionally, a unique molecular type (VNB) of CNSC, a new lineage of CGSC (VGV), and several hybrids between CNSC and CGSC have been found in recent years, all of which were at least partly based on data from PCR fingerprinting (Bovers et al., 2008a; Sidrim et al., 2010; Farrer et al., 2019).

Indeed, PCR fingerprinting using the M13 core sequence as a primer has become a major strain typing tool in the ongoing molecular epidemiological surveys of CNSC and CGSC. Although PCR fingerprinting requires high-quality DNA template and highly standardized conditions to ensure comparability among experiments and labs (Meyer et al., 1999), it combines the specificity of classical DNA hybridization fingerprinting with the speed and simplicity of the PCR reaction (Meyer et al., 1999; Kidd et al., 2004). Furthermore, PCR fingerprinting is of high reproducibility due to the high degree of homology between the primers and the template DNA, which allows the use of high annealing temperatures during the PCR amplification (Meyer and Mitchell, 1995). It is likely that PCR fingerprinting using the M13 core sequence as primer will remain a valuable technique in molecular epidemiological surveys of CNSC and CGSC.

## Amplified Fragment Length Polymorphism

Amplified fragment length polymorphism (AFLP) is a powerful genotyping technique that can discriminate closely related strains and individuals in many groups of organisms, including microorganisms (Vos et al., 1995). This technique can quickly generate a large number of DNA fragments for any organism, with high degrees of reproducibility and discriminatory power (Janssen et al., 1996; Savelkoul et al., 1999; Paun and Schönswetter, 2012). Briefly, AFLP is a PCR-based molecular technique that uses selective amplification of a subset of restriction enzyme digested DNA fragments from any source to generate and compare unique electrophoretic patterns among genomes (Sheeja et al., 2021). Thus, AFLP requires limited amounts of materials but combines the advantages of both RFLP and RAPD in terms of reproducibility and resolution (Janssen et al., 1996; Savelkoul et al., 1999; Paun and Schönswetter, 2012). In addition, it does not require any prior genome information (Sheeja et al., 2021). Overall, despites its higher labor intensity and higher cost than other methods such as RFLP, RAPD and PCR-fingerprinting, the AFLP technique is generally more discriminatory than other molecular typing methods (Savelkoul et al., 1999; Paun and Schönswetter, 2012; Grover and Sharma, 2016). Moreover, several previous studies suggested that the AFLP technique could be used for linkage mapping and for locating genomic regions within the CNSC and CGSC genomes that are related to pathogen virulence, drug susceptibility, and human immune responses (Wiesner et al., 2012; Thompson et al., 2014). Since the 1990s, Boekhout et al. and Hagen et al. have also reported the use of AFLP markers in studies on the taxonomy, molecular epidemiology, genetic diversity, phylogenetic analysis, etc. of the human pathogenic *Cryptococcus* (Kidd et al., 2004; Hagen et al., 2010; Day et al., 2011; Pakshir et al., 2018; van de Wiele et al., 2020).

Similar to PCR fingerprinting, the AFLP technique has been commonly used for epidemiological studies on CNSC and CGSC. Compared to PCR fingerprinting, AFLP has more discriminating power in epidemiological studies of CNSC and CGSC (Ngamskulrungroj et al., 2009; Hagen et al., 2015). For example, a total of 11 AFLP major types have been identified within the human pathogenic Cryptococcus (Hagen et al., 2015), including AFLP1 (CNSC, Serotype A, VNI) (Franzot et al., 1999), AFLP1A (CNSC, Serotype A, VNII/VNB) (Franzot et al., 1999), AFLP1B (CNSC, Serotype A, VNII), AFLP2 (CNSC, Serotype D, VNIV), AFLP3 (CNSC, Serotype AD, VNIII), AFLP4 (CGSC, VGI), AFLP5 (CGSC, VGIII), AFLP6 (CGSC, VGII), AFLP7 (CGSC, VGIV), AFLP8 (CNSC VNIV × CGSC VGI, hybrid) (Bovers et al., 2006), AFLP9 (CNSC VNI × CGSC VGI, hybrid) (Bovers et al., 2008b), AFLP10 (CGSC, VGIV/VGIIIc) (Springer et al., 2014; Trilles et al., 2014), AFLP11 (CNSC VNI × CGSC VGII, hybrid) (Aminnejad et al., 2012). Furthermore, strains’ AFLP patterns have shown overall concordance with results from other strain typing methods such as serotyping, RAPD and PCR fingerprinting among isolates of CNSC and CGSC. For instance, AFLP markers clustered isolates of CNSC and CGSC into the same eight major molecular types as PCR fingerprinting, namely AFLP1 (=VNI), AFLP1A/AFLP1B (=VNII), AFLP2 (=VNIII), and AFLP3 (=VNIV) in CNSC; AFLP4 (=VGI), AFLP5 (=VGIII), AFLP6 (=VGII), and AFLP7 (=VGIV) in CGSC (Meyer et al., 1999; Bovers et al., 2008a; Hagen et al., 2015). AFLP markers contributed to the identification of rare but unique hybrids and to our overall understanding of the diversity and distribution of the human pathogenic *Cryptococcus*. One drawback of AFLP is that DNA banding patterns on gels can be difficult to compare between results from different labs and that fragments of the same size may not be homologous, i.e., containing the same DNA sequences. A summary comparison of results between AFLP with other strain typing methods regarding the major molecular type identifications of the human pathogenic *Cryptococcus* is shown in **Table 2**.

**Table 2:** Summarized classification of CNSC and CGSC based on different molecular markers.

Species complex	Variety	Serotype	PCR fingerprinting	AFLP	IGS1	ITS	Proposed species name	Reference strain	Accession ID of reference assembly
CNSC	var. *grubii*	A	VNI	1	1a/1b	1	*C. neoformans*	H99	ASM301198v1
		A	VNB	1A	1a	1	*C. neoformans*	Bt88	BROAD_CneoA_Bt88_1
		A	VNII	1B	1c	1	*C. neoformans*	PMHc1023.ENR	BROAD_CneoA_PMHc1023.Enr_1
	var. *neoformans*	D	VNIV	2	2a/2b/2c	2	*C. deneoformans*	JEC21	ASM9104v1
	AD hybrid	AD	VNIII	3			*C. neoformans* × *C. deneoformans hybrid*		
CGSC		B/C	VGI	4A	4c	7	*C. gattii*	WM276	ASM18594v1
		B/C	VGI	4B	4a/4b	3/7	*C. gattii*	Ru294	Cryp_gatt_Ru294_V1
		B/C	VGII	6	3	4	*C. deuterogattii*	R265	R265.1
		B/C	VGIII	5A/5C	5	5	*C. bacillisporus*	CA1280	Cryp_gatt_CA1280_V1
		B	VGIII	5B	5	5	*C. bacillisporus*	CA1873	Cryp_gatt_CA1873_V1
		B/C	VGIV	7	6	6	*C. tetragattii*	IND107	Cryp_gatt_IND107_V2
		B	VGV					MF34	Cryp_gatt_MF34

## Multilocus Microsatellite Typing

Multilocus microsatellite typing (MLMT) is a common genotyping method for population genetics and molecular ecology studies of many eukaryotic species, including fungi. MLMT has been used to analyze CNSC and CGSC (De Valk et al., 2007; Karaoglu et al., 2008; Illnait-Zaragozi et al., 2010; Rudramurthy et al., 2011; Pan et al., 2012). Microsatellites (also referred to as short tandem repeats, STRs) are genomic sequences consisting of tandemly repeated short motifs up to 6 nucleotides, which are abundantly present in the genomes of most eukaryotes, including eukaryotic microorganisms. Due to the higher mutation rate of the short repeats during DNA replication (due to strand slippage) than base substitutions, microsatellite markers are especially powerful for analyzing recently expanding populations of organisms (Van Belkum et al., 1998). Microsatellite analysis is usually performed in two steps: (i) amplification of STR loci by PCR and (ii) detection and sizing of amplification products followed by the assignment of repeat numbers. Assignment of repeat numbers is established by comparing the relative electrophoretic mobility of the fragments to the mobility of reference fragments with established repeat numbers (De Valk et al., 2007).

The application of MLMT to analyze CNSC and CGSC samples has resulted in the identification of a large number of genotypes within both CNSC and CGSC (Garcia-Hermoso et al., 2010; Prakash et al., 2020). The power of MLMT in strain discrimination was also shown in a recently emerged human fungal pathogen *Candida auris* where results based on MLMT markers showed similar discrimination power as that based on single nucleotide polymorphisms at the whole genome level (De Groot et al., 2020). Different from RAPD, PCR-finger printing and AFLP, MLMT marker polymorphisms can be directly compared among labs. For example, an MLMT analysis based on 426 Asian clinical CNSC isolates showed a different distribution of genotypes of CNSC isolates from various countries in Asia. The study also identified a correlation between microsatellite genotypes with the original source of strains and their susceptibilities to 5-flucytosine (Pan et al., 2012). Another MLMT analysis of 523 CNSC isolates collected from India showed that environmental isolates were genetically more diverse than clinical isolates (Prakash et al., 2020). However, an MLMT study based on 89 CNSC isolates from Brazil found no differences in antifungal susceptibility and capsule size between major environmental and clinical MLMT types (Zhu et al., 2010). In general, MLMT provides cost-effective genotyping with fast turn-around times and on an individual locus basis, is generally much more discriminatory than MLST. However, there is no global public database for CNSC and CGSC based on MLMT markers. The establishment of such a centralized database would increase its adoption by more researchers and consequently increase our understanding of the recent evolution, gene flow, and epidemiology of the human pathogenic *Cryptococcus*.

## Single Locus and Multilocus Sequence Typing

Revolutions in DNA sequencing techniques have taken the discovery and application of molecular markers to high-throughput and ultrahigh-throughput levels for a variety of studies (Grover and Sharma, 2016). Sequencing single DNA fragments such as the internal transcribed spacer (ITS) region and the intergenic spacer region (IGS) of the ribosomal RNA gene cluster have been broadly used to identify fungal species and strains. For the human pathogenic *Cryptococcus*, ITS, IGS, the *RPR8* gene, and the mitochondrial cytochrome b gene have been commonly used for species and lineage identifications (Diaz et al., 2000; Butler and Poulter, 2005; Diaz et al., 2005). Indeed, ITS region sequence is the universal DNA barcode for fungal species identification, including in many instances for investigating intra-specific variations among strains from different geographic regions and ecological niches (Schoch et al., 2012). Compared to the limited variations in ITS sequences among molecular types within CNSC (Katsu et al., 2004), IGS sequences are more variable with variations represented by nucleotide base substitutions and the presence of long insertions/deletions (indels) (Diaz et al., 2000; Diaz et al., 2005). Some of the differences between IGS and ITS with regard to variations in CNSC are shown in **Table 2**. The pros and cons of using single locus sequences for species and strain identifications have been extensively discussed (Xu, 2016; Xu, 2020).

The limited variation at an individual locus such as ITS and IGS within some species can be overcome by sequencing multiple loci located in different parts of the genome. Indeed, MLST is a commonly used method for genotyping strains of many common human microbial pathogens. With the use of sequence information from multiple loci, MLST can provide high discriminatory power and allow reproducible results to be shared among laboratories. The first MLST study of the human pathogenic *Cryptococcus* was published in 2000 (Xu et al., 2000b). Since then, MLST has been applied to many studies on the epidemiology of CNSC and CGSC (Sugita et al., 2001; Taylor and Fisher, 2003; Fraser et al., 2005; Litvintseva et al., 2006). For example, both single locus and multilocus sequence typing of serotype AD strains identified that these strains were recent hybrids and revealed evidence for sexual reproduction in natural populations of both the VNI (serotype A) and VNIV (serotype D) lineages within CNSC (Xu et al., 2002; Xu and Mitchell, 2003).

Early MLST studies of the human pathogenic *Cryptococcus* often sequenced different loci and resulted in inconsistent numbers and nomenclatures of those sub-groups (Sugita et al., 2001; Bovers et al., 2008b). Consequently, there were difficulties in comparing epidemiological results among studies. In 2009, the International Society for Human and Animal Mycology (ISHAM) *Cryptococcus* Working Group agreed upon MLST using seven loci: *CAP59*, *GPD1*, *LAC1*, *PLB1*, *SOD1*, *URA5* and *IGS1* region as the standardized genotyping approach (Meyer et al., 2009). The application of a standardized set of loci for sequence typing of strains led to an expanding dataset and the establishment of a reference database from which future studies could continuously build on (https://mlst.mycologylab.org/). MLST analyses based on the shared DNA sequences provided a comprehensive view on the global distribution of genotypes (Litvintseva et al., 2006; Bovers et al., 2008b; Ngamskulrungroj et al., 2009), including unique clades and sub-clades within CNSC and CGSC in specific regions in the world (Chowdhary et al., 2011; Beale et al., 2015). For example, MLST analysis detected higher genetic diversity in the South African *C. neoformans* var. *grubii* isolates than those from other geographic regions, which led to the ‘Out of Africa’ hypothesis for the origin and dispersal of *C. neoformans* var. *grubii* around the globe (Litvintseva et al., 2011; Simwami et al., 2011; Litvintseva and Mitchell, 2012). In addition, MLST analyses identified that the East Asian clinical population of *C. neoformans* var. *grubii* was highly clonal and dominated by one sequence type ST5 and its closely related sequence types (Simwami et al., 2011; Khayhan et al., 2013; Dou et al., 2015; Fan et al., 2016; Day et al., 2017; Chen et al., 2018; Hong et al., 2019). For CGSC, comparisons of genetic diversity of isolates from different locations based on MLST analyses indicated northern Brazil was likely the source for the global expansion of the VGII lineage, including for strains causing the outbreak in the Pacific Northwest of North America (Hagen et al., 2013; Souto et al., 2016), and revealing a possible introduction of Australian VGII into Vancouver Island in western Canada (Fraser et al., 2005; Byrnes et al., 2009; Byrnes et al., 2010). Moving forward, MLST will continue to be a major strain typing method for epidemiological studies of the human pathogenic *Cryptococcus.*

## MALDI-TOF MS

Aside from the DNA fragment-based sequencing and targeted protein-based molecular strain typing methods described above, there is another common molecular method for identifying fungal species called matrix-assisted laser desorption/ionization time of flight mass spectrometry (MALDI-TOF MS). This method is based on species-specific spectra of the masses of peptides and proteins in colonies of microbial cells. It was first developed for bacterial identification (Claydon et al., 1996; Krishnamurthy and Ross, 1996) and subsequently extended to fungal identification (Li et al., 2000; Van Veen et al., 2010; Alshawa et al., 2012; De Carolis et al., 2012). MALDI-TOF MS has become a rapid, easy-to-use, and inexpensive method for identifying clinically important microorganisms (Claydon et al., 1996; Krishnamurthy and Ross, 1996; Seng et al., 2009). Indeed, this technology has been used to identify hundreds of CNSC and CGSC strains isolated from both humans and animals (Firacative et al., 2012; Posteraro et al., 2012; Jin et al., 2020; Zvezdanova et al., 2020; Florek et al., 2021). In a recent study, an expanded database allowed the MALDI-TOF MS technology to separate the two major lineages within CNSC, VNI (*C. neoformans*) and VNIV (*C. deneoformans*), including identifying most of their hybrids (VNIII) when the authors used a hierarchical clustering approach and focused on five selected biomarkers in the dataset (Zvezdanova et al., 2020). However, the reproducibility of MALDI_TOF MS for either lineage or strain identifications have not been critically evaluated by different labs for the same set of strains.

In addition to (potentially) allow direct identification of pathogens based on protein profiles, MALDI-TOF MS holds great promise for diagnosing and genotyping pathogenic fungal species based on other cellular molecules. For example, due to their essential roles in cell integrity, growth, and reproduction, lipids such as membrane phospholipids are found in all cellular organisms, including strains of CNSC and CGSC. However, different fungal species and strains can differ in their lipid profiles, thus making lipids a promising group of molecules to potentially serve as biomarkers for taxonomic and metabolic characterization of fungal pathogens (Stübiger et al., 2016). For example, because fungal membrane lipids such as ergosterol are targets of several commonly used antifungal drugs, lipid analysis can potentially help identify medically important features such as antifungal resistance among clinical strains, thus benefiting patient treatments. Overall, due to its simplicity, high efficiency and increasing availability, the MALDI-TOF MS technology can be a valuable tool for molecular lineage identification within CNSC and CGSC, and thus representing a potential future alternative for screening drug susceptibilities among strains.

## Whole-Genome Sequence Typing (WGST)

With rapidly falling cost, whole genome sequencing typing (WGST) has been applied for epidemiological studies of a variety of organisms, including for CNSC and CGSC. Compared to epidemiological studies of CNSC and CGSC utilizing other strain typing methods, whole-genome sequencing is capable of capturing the complete genetic variation, including single nucleotide polymorphisms, insertions and deletion, gene copy number variations, and genome structural rearrangements. However, to reveal all of the above genetic variations, very high sequence coverages employing sequencing platforms capable of generating both short and long sequence reads are needed. At present, most epidemiological surveys of microbial pathogens using the whole-genome sequencing approach rely on the Illumina MiSeq or HiSeq platforms to obtain short-read DNA sequences. Such short reads at greater than 50X coverage are generally sufficient for robust identification of SNPs for inferences of strain relationships and epidemiological patterns (Beale et al., 2015; Cuomo et al., 2018).

However, for most genomic epidemiological comparisons to be informative, well-annotated reference genome assemblies are generally needed. Fortunately, within the human pathogenic *Cryptococcus*, reference genomes representing *C. neoformans* var. *grubii* (VNI, VNII, VNB) and var. *neoformans* (VNIV), and each of the five molecular types of CGSC (VGI, VGII, VGIII, VGIV and VGV) are available (Fraser et al., 2005; Galagan et al., 2005; Loftus et al., 2005; D’souza et al., 2011; Janbon et al., 2014; Farrer et al., 2015; Rhodes et al., 2017a). Indeed, to date, three *C. neoformans* var. *neoformans* genomes, 58 C*. neoformans* var. *grubii* genomes and 7 CGSC genomes have been assembled and annotated, providing nearly complete catalogs of genes within both CNSC and CGSC (**Table 2**). These genome assemblies provide genomic resources for the community and enable a wide array of downstream studies. Genome sequence comparisons between different molecular types of CGSC revealed a sequence divergence of >3% among the major lineages and molecular types (D’souza et al., 2011; Farrer et al., 2016). In addition to the average nucleotide identity in among the genomes, there were several other notable findings. For example, gene structure comparisons revealed that genes of CNSC have more introns with more alternative splicing (Loftus et al., 2005; Janbon et al., 2014) than those of CGSC (Farrer et al., 2016). In addition, strains of the VGII clade responsible for the cryptococcal outbreak on Vancouver Island seemed to have lost the genes involved in RNA interference (Loftus et al., 2005; Janbon et al., 2014). Furthermore, these well-annotated reference assemblies have enabled the construction of functional genomic resources, such as a deletion collection of *C. neoformans* var. *grubii* genes in the H99 strain background (Liu et al., 2008). Such resources make it easier for the community to studying the relationships between specific genes and phenotypic differences between strains and populations.

In addition to revealing the similarities and differences among natural strains from within and between different lineages, whole genome sequencing can help identify microevolution of infecting strains within patients by comparing cryptococcal genomes at initial presentation and later such as during relapse of infection (Ormerod et al., 2013; Chen et al., 2017; Rhodes et al., 2017a). For example, in one study, the deletion of a transcriptional regulator and changes in the copy number of genes on chromosome 12 were found between a pair of initial and relapse isolates, with such changes correlated with marked virulence-related phenotypic differences between the strains (Ormerod et al., 2013). Similarly, based on genome sequencing of 38 initial and relapse isolates from 18 patients, specific mutations were found in genes involved in growth at 39°C, stress response, and capsule production (Chen et al., 2017). Indeed, microevolution studies have revealed that clinical isolates are capable of rapid adaptation through hypermutation, frequently due to mutations in mismatch repair gene *MSH2* in CGSC (Billmyre et al., 2014; Billmyre et al., 2017) and CNSC (Boyce et al., 2017; Rhodes et al., 2017a).

With the increasing availability of whole genome sequencing, both CNSC and CGSC have now had hundreds of individual isolates sequenced, providing fine-scale insights into their evolution and diversification (Desjardins et al., 2017; Rhodes et al., 2017b; Vanhove et al., 2017; Ashton et al., 2019). Population genomic methods, such as principal component analysis (PCA), admixture analysis and phylogenetic analysis based on whole genome sequencing, are playing an increasingly important role in clustering cryptococcal isolates and tracing the origin of individual strains and/or groups of strains (Billmyre et al., 2014; Engelthaler et al., 2014; Desjardins et al., 2017). In combination with other methods, population genomics can help identify genes related to the evolution of virulence and pathogenicity, including putative novel drug targets (Farrer et al., 2015; Desjardins et al., 2017).

For CGSC, genome-level phylogenetic studies have suggested VGII strains, the main strains responsible for the cryptococcal outbreak in the Pacific Northwest of North America, can be divided into three main subgroups: VGIIa, VGIIb and VGIIc. Furthermore, the analyses provided evidence that the main outbreak lineage might have an origin in South America (Billmyre et al., 2014; Engelthaler et al., 2014). In addition, whole-genome comparisons recently revealed a new lineage of CGSC isolates (VGV) in Zambia (Farrer et al., 2019). For CNSC, a recent population genomic study found evidence for two sub-lineages within VNB, VNBI and VNBII. Such a finding helped reveal phenotypic differences between these two subgroups (Desjardins et al., 2017). Though there were some differences, results from mitochondrial exon sequence analyses were largely consistent with the existence of two subgroups VNBI and VNBII within VNB, as inferred based on nuclear genome SNPs (Wang and Xu, 2020). However, the distributions of mitochondrial introns were quite mixed within CNSC, suggesting their frequent gains and losses during evolution (Wang and Xu, 2020). Several population genomic studies have found a highly clonal cluster of VNI isolates capable of infecting HIV-negative patients in east Asia (Day et al., 2017; Ashton et al., 2019). Multidrug transporters, aconitases (iron-sulfur proteins), capsule genes, heat-shock proteins and protein kinases were found to be under positive selection in multiple sub-lineages of CGSC, which suggested that these genes might play an important role in the adaptation of CGSC isolates to host environments (Farrer et al., 2015; Farrer et al., 2016). Finally, the large number of publicly available genomes provides a fertile ground for genome-wide association studies (GWAS) between genetic variants and phenotypic differences among strains. A recent GWAS study found that the loss-of-function mutation in the transcription factor *BZP4* was linked to melanization capacity among VNB isolates (Desjardins et al., 2017).

Overall, epidemiological studies utilizing whole genome sequencing have significantly improved our understanding of genetic divergence between lineages, gene flows across geographic regions, and gene gains and losses during the evolution of different lineages within both CNSC and CGSC. However, as mentioned above, most currently available genome sequences of CNSC and CGSC strains were generated using the short-read Illumina platforms, the quality and utility of which could be affected by sequencing depth, library preparation chemistry, and sequencing bias (Rhodes et al., 2014). Previous studies have shown that most cryptococcal assemblies generated from the Illumina platform contain many sequencing gaps, making it difficult to infer chromosomal structural polymorphisms in these genomes (Day et al., 2017; Rhodes et al., 2017b). In the future, the incorporation of long reads generated by Pacific Biosciences or Oxford Nanopore Technologies will allow more complete genome assemblies and provide robust foundations for other types of genomic comparisons such as chromosomal reversions and translocations (Van Der Straaten, 2015; Cuomo et al., 2018).

## Conclusions and Future Directions

Our literature review above showed that many molecular techniques have been successfully used for *Cryptococcus* epidemiological studies. Over the past 30 years, we have seen a rapid development of strain typing methods to differentiate species and strains of this group of important human fungal pathogens (**Figure 1**). These studies have helped resolve species boundaries, identify hybrids, reveal both local and global genetic diversities and gene flows, and pinpoint specific mutations accumulated during the microevolution of strains within patients. As shown above, all techniques have helped improve our understanding of cryptococcal epidemiology and each technique has its advantages and limitations (**Table 1**). For example, though relatively crude, PCR fingerprinting and AFLP analysis established the most widely used taxonomy and nomenclature system for CNSC and CGSC. Though there are still controversies (Hagen et al., 2015; Hagen et al., 2017; Kwon-Chung et al., 2017), the applications of more precise molecular typing methods, especially WGST have moved the taxonomy and nomenclature system to become increasingly clear for the human pathogenic *Cryptococcus*. Resolution of the controversies in these organisms could potentially serve as a model for other fungal species (Xu, 2020). For cryptococcal epidemiological studies, we expect the increasing use of MLST, MLMT, MALDI-TOF MS, and WGST in the future with different methods serving slightly different but complementary purposes.

## Author Contributions

Writing—original draft preparation: NH, MC, and JX. Writing—review and editing: JX. All authors contributed to the article and approved the submitted version.

## Funding

This review was funded by National Natural Science Foundation of China (grant numbers 81201269 and 81720108026), Innovation Team Foundation of Jiangsu Province (grant number CXTDA2017038), National Medicine Foundation of China (grant number CLB20J022), Natural Science and Engineering Research Council of Canada (grant number RGPIN-2020-05732), and McMaster University (Global Science Initiative-2020-03).

## Conflict of Interest

The authors declare that the research was conducted in the absence of any commercial or financial relationships that could be construed as a potential conflict of interest.
